# Exfoliation and physico-chemical characterization of novel bioplasticizers from *Nelumbo nucifera* leaf for biofilm application

**DOI:** 10.1016/j.heliyon.2023.e22550

**Published:** 2023-11-20

**Authors:** Divya Divakaran, Malinee Sriariyanun, Indran Suyambulingam, Sanjay Mavinkere Rangappa, Suchart Siengchin

**Affiliations:** a Natural Composites Research Group Lab, Department of Materials and Production Engineering, The Sirindhorn International Thai-German School of Engineering (TGGS), King Mongkut's University of Technology North Bangkok (KMUTNB), Bangkok, 10800, Thailand; b Biorefinery and Process Automation Engineering Center, Department of Chemical and Process Engineering, The Sirindhorn International Thai-German Graduate School of Engineering, King Mongkut’s University of Technology North Bangkok, Bangkok, 10800, Thailand

**Keywords:** *Nelumbo nucifera* leaf, Polymers, Bio-plasticizers, Extraction, Characterization, Composites

## Abstract

Due to the extreme threats as environmental and health issues caused by the petroleum-based leachable plasticizers, researchers among different domains are more interested in finding unique biodegradable plasticizers from natural sources. The present study used *Nelumbo nucifera* leaf to extract novel biopolymers as viable substitutes for chemical plasticizers. The biopolymers extraction was carried out through chemical means and its physico-chemical and morphological characterization were carried out to confirm its plastic nature. The polymers extracted possess a low glass transition temperature (77.17 °C), good thermal stability (230 °C), low density (0.94 g/cc), good surface roughness (34.154 μm), low crystallinity index (25.1%) and moderate crystallite size (16.36 nm). The presence of an organic polymer with specific chemical groups as olefinic alkenes, epoxide, imino/azo groups, and hydrophobic organic siloxane groups, signify that the material is a condensed phenolic derivative. Furthermore, bio-film was formulated using NLP and poly lactic acid (PLA) matrix to evaluate its plasticizing effect and film-forming ability. Variation in specific properties of film was noted after bio-plasticizer addition, where tensile strength (20.94 ± 1.5 MPa to 19.22 ± 1.3 MPa) and Young's modulus (1.462 ± 0.43 GPa to 1.025 ± 0.52 GPa) was found to be decreased whereas increased the percentage of elongation at break (26.30 ± 1.1% to 39.64 ± 1.6%). In addition, decreased glass transition temperature (Tg) (59.17 °C), good surface compatibility, and increased flexibility of NLP-PLA film in contrast to pure PLA film authorizes the plasticizing effect of bio-plasticizers on PLA. Since the extracted bio-plasticizers could be a suitable replacement to harmful synthetic plasticizers for lightweight packaging applications in bioplastics sector.

## Graphical abstract

**Figure:**
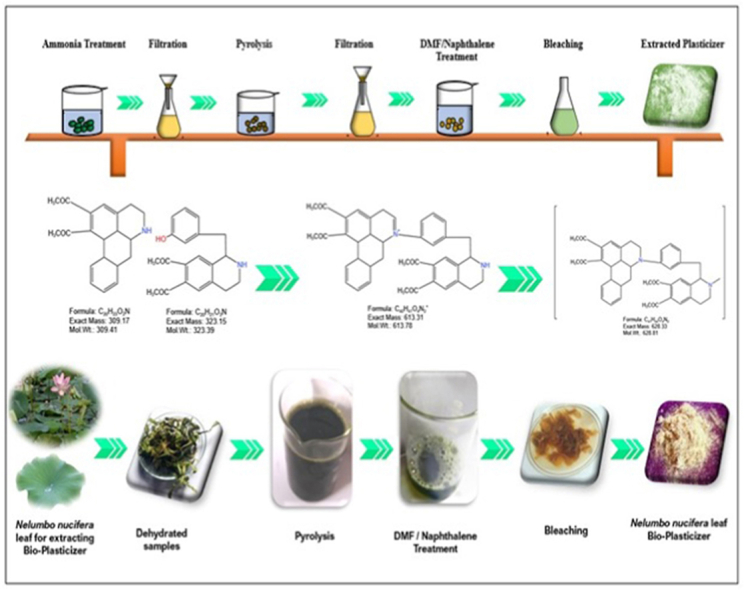

## 1 Introduction

Nowadays, research and developments are desired to explore potential, bio-degradable, cost-effective, and eco-friendly green materials that replace existing, detrimental, non-disposable synthetic ones by compromising environmental standards [1]. Bio-plasticizers can meet such criteria along with offering sustainable production due to their renewable nature. The main advantages of using bio-plasticizers are renewability, degradability, light-weight nature, cost effectiveness, good miscibility, high resistance to leaching, environmentally friendliness, easy processability, and non-hazardous nature [2]. Adding a bio-plasticizer to a polymer system creates more room in the matrix, which improves the material's pliability, workability, and distensibility [3]. Further, this material has a low atomic weight and can reduce the glass transition temperature of a different material that has a higher Tg [4,5]. Bio-plasticizers can be employed in best possible ways through copolymerization as well as incorporating it with other polymers as blends. Previous reports indicated improved mechanical, thermal, and rheological properties in polymer mixtures. Even though, the development of renewable, eco-friendly, lightweight, degradable but long- durable green composite material and its property enhancement is the major challenge of composite research today [6,7].

The typical synthetic phthalate plasticizer-based composites or films exhibited plasticizer migration or diffusion that further leads to disagreeable property changes. This causes lethal effects on human health and environment [8,9]. However, bio-plasticizers partakes unique physical, chemical, thermal, and surface properties and possess biocompatibility and film-forming ability too. They can be used to synthesize biodegradable, lightweight, renewable materials that ensure good mechanical properties. Due to the extended functionality of bio-plasticizers over synthetic phthalates ones, the plastic industry is being replacing traditional plasticizers [4,10]. A forecast revealed that the demand of bio-plasticizers would be increasing 9.75 million tonnes in 2024 as mentioned in the Cabot Corp report, 2017. According to a report by Grandview Research, the bio-plasticizers market could reach $268.37 million by 2025, up from $112.43 million in 2016 [11]. When it comes to the development of lightweight materials appropriate for all industries—and especially for food packaging—bio-plasticizer based composites or bio-films may prove to be an attractive alternative to synthetic phthalate-based materials [12,13].

Plants are the major sources of biopolymers, which synthesize secondary metabolites or biopolymers from primary metabolites through a wide range of metabolic reactions [14,15]. A wide range of agricultural resources can synthesize bio-plasticizers, which includes cereals, fruits, vegetables, oleaginous plants, trees, etc. [4,[16], [17], [18]]. The utilization of accessible plant sources or residues for bio-plasticizer extraction is another overwhelming strategy that is highly economic and eco-friendly and would assist sustainable development along with reducing the other environmental problems as global warming, residue/waste management, toxic release, etc. [[19], [20], [21]]. Although, the identification of potential biomass sources for bio-plasticizer extraction is of having much interest nowadays [19]. These bio-based plasticizers, which come in epoxidized, ester, or glyceryl form, have the potential to compete with *o*-phthalate plasticizers derived from petroleum [22]. Considering this, the main goals of the suggested work were to extract and characterize the bio-plasticizers from *Nelumbo nucifera* leaves and investigate whether the bio-plasticizers could be used to fabricate biofilms [6,23,24].

The aquatic plant *Nelumbo nucifera* (from the family *Nelumbonaceae*) is commonly grown in ponds and other small bodies of water. Their range includes East Asia, China, Russia, the Caspian Sea, Australia, and more [25]. There is always a supply of *Nelumbo nucifera* because it is a perennial plant that multiplies rapidly. After four months of cultivation, it reaches full yield in water depths of up to eight feet. The plant has big, round leaves that measure about 80 cm across. These secondary metabolites may possess plasticizing effect and therefore, exploration of such renewable, easily accessible, eco-friendly, biodegradable bio-plasticizers eliminates the detrimental issues linked with synthetic plasticizers [6]. Moreover, there were no significant uses reported for secondary metabolites of *Nelumbo nucifera* leaves as bio-plasticizers, as of now.

Regardless of these realities, more progress is necessary to make use of this kind of available biomass to guarantee the sustainable socioeconomic development of each country [[26], [27], [28], [29]]. Thermo-chemical treatment has a greater impact on isolating secondary metabolites or biopolymers from the biomass and further characterization is necessary to confirm the characteristics of the biopolymers in terms of their chemistry, temperature, and topography [[30], [31], [32]]. Since, bio-polymers from *Nelumbo nucifera* leaves were extracted through thermo-chemical method and newly isolated bio-polymers were comprehensively characterized to establish its suitability for bio-film formulation. For potential future uses, the plasticizing effect of isolated bio-plasticizers was investigated by reinforcing them with a PLA matrix and comparing its properties to those of other well-known plasticizers.

## 2 Materials and methods

### 2.1 Selection of a sustainable agro-waste source

The foremost step of current research was the selection of a potential plant material for the isolation of bio-plasticizers. The present study chosen the leaves of *Nelumbo nucifera* for the extraction of biopolymers based on certain criteria’s as easy accessibility, sustainability, environmental concerns, and easy processability. A pond system in Asarivilai, Kanyakumari district, Tamil Nadu, India, provided the required quantity of *Nelumbo nucifera* leaves.

### 2.2 Extraction of bio-plasticizers

**Fig. 1:**
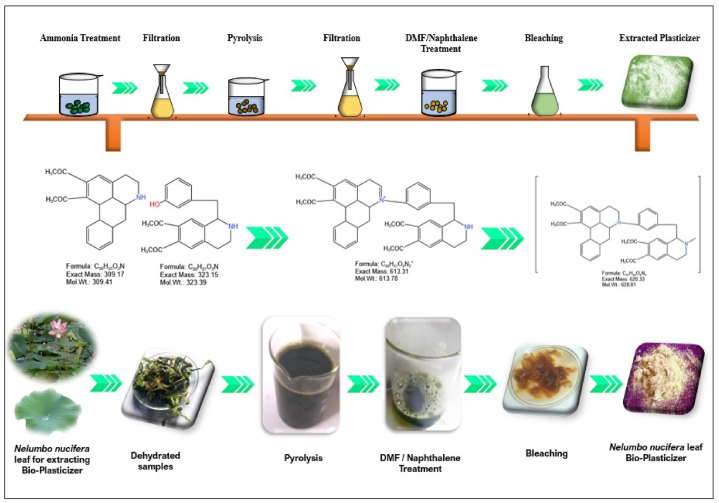
Preparation stages of bio-plasticizers from *Nelumbo nucifera* Leaf.

The raw material collected was primarily treated with ammonia to remove water, minerals and toxic contents that were attached to the surface, if any. Followed by the dried samples were subjected for thermo-chemical treatment coupled with acids to accomplish slow chemical pyrolysis of the sample. This process is carried out in a laboratory scale closed glass beaker system, where an appropriate amount of raw material is placed inside along with 5% concentrated potassium chloride and hydrochloric acid solution for 4–5 h at ambient temperature. Pyrolysis allowed the conversion of samples to semi-solid state that possess high carbon content with less liquid or gases. The obtained solid residue was further treated with dimethyl formamide (DMF) and acetone to separate pigments, minerals, primary and secondary metabolites. The resultant supernatant was then treated with naphthalene to release derivatives of primary metabolites as flavonoids, phenolic compounds and glucosides (secondary metabolites) that possess plasticizing effect. Bleaching with sodium hypochlorite separated and purified the supernatant plasticizers. Since wastes have different chemical compositions, bio-plasticizer extraction from biomass requires optimization. The time of exposure of chemicals vary according to the concentration of lignin, pigments and other unwanted elements that present in the material. On the other hand, repeated purification steps may require if the extracted material contains more pigments or colored derivatives. Fig. 1 displays the plasticizer extraction steps followed in the present study.

### 2.3 Characterization of NLP bio-plasticizers

The extracted bio-plasticizers' physicochemical, thermal, and surface properties were analyzed using standard protocols to determine the material's specific features. Physical analysis is required to determine the specific density of the material, which is necessary to disclose the fitness of bio-plasticizer for light-weight application. The chemical analysis was helpful in determining the major chemical constituents, functional groups, and crystallinity of the substance under investigation. Thermal testing and morphological analysis were performed to establish the temperature endurance and surface qualities of the extracted biopolymers and whether they were suitable for composites or biofilms [33].

#### 2.3.1 Density analysis of NLP

An AccuPyc II 1340 model pycnometer measured the density of NLP plasticizers while helium gas flowed. Prior to subjecting, the sample was dried in an oven at 105 °F for 24 h to eliminate the moisture content. The samples were then kept in a desiccator to ensure complete eradication of water. After five measurements at room temperature (27 °C), the mean density was calculated using equation (1). Where, ‘*m’* indicates mass in grams and ‘*V’* represent the volume (cm^3^) [34,35].

(1) Density=mV

#### 2.3.2 FT-IR analysis

NLP functional groups were identified using a Jasco 4600 Fourier transform infrared analyzer. The sample container correctly held the powdered material, and the experiment was done in non-contact mode with 4 cm^−1^ resolution. After that, the FT-IR spectra were recorded at a temperature of around room temperature, focusing on the wavelength range of 4000–500 cm^−1^. The resultant spectrogram was further used to explain the chemical groups and associated chemical components inhabited in the suspicious material, in order to verify its chemical make-up and plasticizing nature.

#### 2.3.3 NLP X-ray diffraction analysis

The crystalline and amorphous portions of the NLP were found through the use of XRD analysis. The experiment was carried out using an X-ray diffraction system manufactured by Bruker. Before the X-rays were shielded, the extracted samples were organized for examination by being placed in the sample holder. The X-ray generator was set to operate at a current of 10.0 mA and a voltage of 30.0 kV. Followed by X-rays of 1.54060A° were shielded on the material and the diffracted X-rays were detected while shifting the detector from 2θ = 20°–80°, at a scanning rate of 0.020°/80 s. An ideal temperature of 25 °C was maintained throughout the system.

(2) $$\text{CI}\left(\%\right)=\frac{A_{\text{crystalline}}}{A_{\text{crystalline}}+A_{\text{amorphous}}}\times 100$$

(3) $$D=\frac{k\lambda }{\beta \;\cos\;\theta }$$

The crystallinity index (CI) and crystallite size (CS) of the biopolymers were determined with the help of the Origin Pro 2021 software. These values were obtained by applying equations (2), (3). In equation (2), the crystalline curve fraction is denoted by the symbol Acrystalline, and the amorphous curve fraction is represented by the symbol Aamorphous. Scherrer's formula can be expressed as equation (3), where the constant 0.89 of Scherrer is denoted by K, the specific wavelength is represented by, (CUKα = 1.5406°A) is D is the crystalline size, and Bragg's angle is the line's full width at half-maximum wavelength (nm).

#### 2.3.4 UV visible spectroscopy analysis of NLP

In order to analyze the isolated biopolymers, a Shimadzu UV-2101 PC spectrometer and an integrating sphere were utilized. Recordings were made of the spectrum observable to the human eye from 200 to 700 nm. Spectroscopic peaks are irrefutable evidence that plasticizing ingredients are present [36].

#### 2.3.5 Thermal analysis

TGA and DSC were utilized in order to explore the thermal profiles of the bio-plasticizers that were separated for the purposes of this investigation. The TGA test was necessary in order to demonstrate thermal stability, however the DSC analysis was sufficient in order to determine the temperature at which glass transitions occur (Tg) [37,38].

##### 2.3.5.1 Thermogravimetric analysis (TGA)

TG/DTA Exstar 6300 was used to understand the thermal profile in terms of thermal stability and degrading features of NLP. The required sample was loaded in an alumina crucible and the examination was conducted in a nitrogen-rich inert environment. The sample was heated from 30 °C to 800 °C at 20 °C/min. Various important intervals of weight loss were recorded to assess the link between mass loss and heating temperature. Decomposition rates of various chemical residents of the sample at different temperatures could be determined with reasonable accuracy using differential thermographic (DTG) analysis [39]. The NLP's kinetic activation energy was calculated using the Coats-Redfern equation (4); [40].

(4) $$\ln\left[\frac{g\left(x\right)}{T^{2}}\right]=\ln\left[\frac{AR}{\beta E}\left(1-\frac{2RT}{E}\right)\right]-\frac{E}{R}\frac{1}{T}$$

where T is the reaction time in minutes, R denotes the universal gas constant (8.314 J mol^−1^ K^−1^), t specifies the duration of reaction (Kelvin), β $\left(\beta \frac{dt}{dx}\right)$ is the linear heating rate and the integral form of the reaction mechanism is given as g (x).

##### 2.3.5.2 Differential scanning calorimetry (DSC)

The DSC analysis was conducted to establish the glass transition temperature (Tg) of the NLP extracted. A differential scanning calorimeter model Mettler STAR^e^ SW 16.20 was used for the purpose, where a suitable quantity (3.5 mg) of powdered sample and a control were loaded to the sample holder in an alumina crucible. A heating rate of 20 °C/min was used to raise the sample temperature from 0 °C to 400 °C. The resultant spectrogram was used for detecting the specific glass transition temperature of the material, and thereby to confirm the plasticizing nature of the material [41,42].

#### 2.3.6 Surface property analysis

To prove the extracted material is suitable for composite or biofilm applications, bio-plasticizer surface fineness, components distribution, and surface characteristics must be shown. EDX, FE-SEM, and AFM were utilized to study bio-plasticizers' surface properties (SEM-EDX) [43].

##### 2.3.6.1 SEM/SEM-EDX analysis

Using a Tescon Vega (third-generation) SEM analyzer, we were able to better understand the surface morphology of NLP. The accelerating voltage used in the test was 5 kV. The SEM images were taken at various magnifications, including 500X, 1000X, and 1500X [44]. Particle size distribution of the material was evaluated from the images using the software Image J [45]. The average size of the particles was measured by calculating the size of 21 particles. The imperative elements on the surface of NLP were identified by employing an EDX analyzer model INCAPentaFETx3 along with a Tescon Vega scanning electron microscope. The EDX spectra were collected from specific spots and reported [46].

##### 2.3.6.2 AFM analysis

The surface characteristics of the NLP was established with the aid of a Flex AFM instrument, in order to validate SEM findings. In order to create topographical images of the NLP surface, the device's scan head resolution was set to 0.1 nm. In addition, the obtained images were planned to be used for calculating the essential surface parameters of bio-plasticizers, such as the total roughness profile, average roughness, roughness skewness, roughness kurtosis, root mean square deviation, surface peak-to-valley difference, etc. The obtained roughness parameters are further discussed to reveal its efficiency for bio-composite of biofilm design with other polymers.

### 2.4 Analysis of plasticizing effect of NLP with PLA

Bio-film was prepared with NLP and polylactic acid (PLA) to analyze the plasticizing effects of NLP reinforcement (Fortunati et al., 2015). Previous studies have reported that a concentration of 2.5–5% plasticizers showed notable variations in diverse properties of bio-films, however, high concentrations (10–30%) resulted substantial variation in specific properties [47]. The focus of this study was to assess the bio-compatible nature and film-forming ability of NLP with PLA. The present study had assessed the compatibility of NLP with PLA matrix by using a concentration of 5% NLP only. PLA was dissolved in chloroform (CHCl_3_) using a magnetic stirrer at room temperature for 1 h to make matrix (Fortunati et al., 2015). Solvent casting produced pure PLA (control) and 5% NLP-PLA films. The PLA solution was homogenized by adding the required amount of NLP filler and stirring with a magnetic stirrer for 1 h. Control PLA film was made without NLP. The viscous solution was cast onto a 150mm × 50 mm petri plate and dried at ambient temperature for 12 h in a desiccator. The films were removed from the petri plates and tested.

A Quro QRS-S11H universal testing machine were used to analyze the bio-film tensile strength, modulus, and elongation at break. The flexibility of the film was checked by naked eye, and morphological studies were done using a Tescon Vega SEM analyzer to confirm NLP's reinforcing property with PLA. Previous studies showed that bio-plasticizers lower polymer material glass transition temperature (Tg), so the film's Tg was measured to confirm the NLP's plasticizing effect.

## 3 Results and discussion

### 3.1 Physical property of NLP

The bio-plasticizers obtained from *Nelumbo nucifera* leaf has a low density of *0.94* g per cubic centimeter. The measured density was comparable with the density of other synthetic and bio-based plasticizers, in such case, the density has been reported to be in the range of 0.93–1.209 g/cc. The density noted for NLP was almost closer to that of DPHP, Diundecyl phthalate, Ditridecyl phthalate, Bis (2-ethylhexyl) adipate, and Dibutyl sebacate (Table 1). Although, relatively low density noted for the *NLP* shows its potential in fabricating light-weight bio-composite and biofilms by incorporating into other polymers.

**Table 1:** Density of *NLP* in comparison with other phthalate and bio-based plasticizers.

Sl. No	Plasticizer	Density	Reference
1	*NLP*	*0.*94 g/cc	*(Current work)*
2	*Dimethyl phthalate*	*1.*191 g/cc	[48]
3	*Diisobutyl phthalate*	*1.*038 g/cc	[49]
4	*Di-n-hexyl phthalate*	*0.*995 g/cc	[50]
5	*Dibutyl phthalate*	*1.*05 g/cc	[51]
6	*Benzyl butyl phthalate*	*1.*119 g/cc	[52]
7	*Emoltene*	*0.*960 g/cc	[50]
8	*Bis (2-ethylhexyl) phthalate*	*0.*99 g/cc	[52]
9	*Diisononyl phthalate*	*0.*98 g/cc	[52]
10	*Diisononyl phthalate*	*0.970-0.*977 g/cc	[50]
11	*DPHP*	*0.957-0.*965 g/cc	[53]
12	*Diisodecyl phthalate*	*0.96-0.*97 g/cc	[54]
13	*Diundecyl phthalate*	*0.*954 g/cc	[50]
14	*Dioctyl terephthalate*	*0.*984 g/cc	[55]
15	*Ditridecyl phthalate*	*0.953-0.*959 g/cc	[50]
16	*Bis (2-ethylhexyl) adipate*	*0.*93 g/cc	[56]
17	*Benzyl phthalate*	*1.*090 g/cc	[50]
18	*Sebacic acid*	*1.*209 g/cc	[57]
19	*Di-(2-ethyl hexyl) phthalate*	*0.980-0.*985 g/cc	[50]
20	*Di-(2-propyl heptyl) phthalate*	*0.960-0.*985 g/cc	[50]
21	*Dibutyl sebacate*	*0.*9405 g/cc	[57]

### 3.2 Chemical functional groups of NLP

**Fig. 2:**
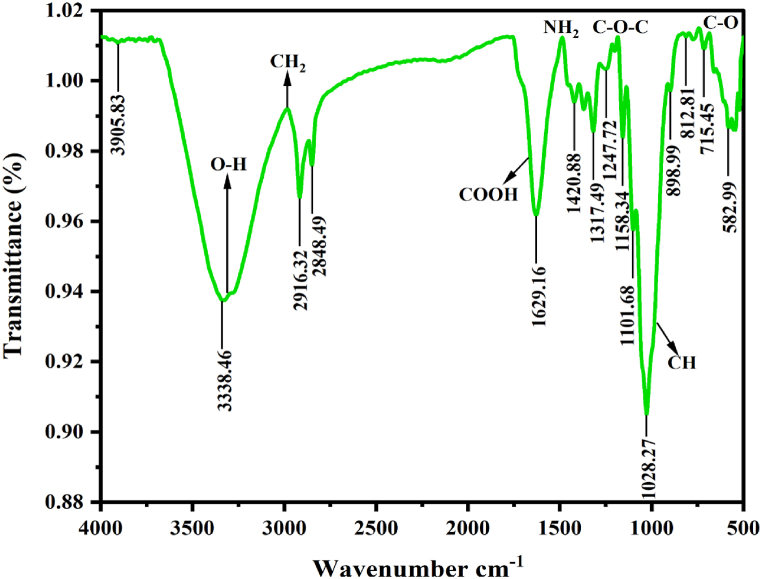
FT-IR spectrogram of NLP.

Fig. 2 presents the FT-IR spectrogram of newly isolated plasticizer (NLP) and each of the peaks in the spectrogram represent a specific functional group existing on it. The identification of hydrogen bond interaction could be probable by analyzing the FT-IR spectra obtained. Spectra with more than five absorption bands indicate that the material is a complex compound with high molecular weight, not an organic compound or inorganic salt [57]. There are 14 attributions noted in the spectra at 3338.46 cm^−1^, 2916.32 cm^−1^, 2848.49 cm^−1^, 1629.16 cm^−1^, 1420.88 cm^−1^, 1317.49 cm^−1^, 1247.72 cm^−1^, 1158.34 cm^−1^, 1101.68 cm^−1^, 1028.27 cm^−1^, 898.99 cm^−1^, 812.81 cm^−1^, 715.45 cm^−1^, and 582.99 cm^−1^. The foremost strong, broad, bending vibration noted at 3338.46 cm^−1^ indicates that the material possesses O–H and N–H functional groups, which revealed that the material is a polymeric compound that contain alcohol and aliphatic amines. Generally, bio-plasticizers are bio-polymers that made-up of polymeric cyclic structures or phenolic compounds attached with hydroxyl and other chemical groups. The following two small, sharp stretches obtained at 2916.32 cm^−1^ and 2848.49 cm^−1^ indicates C–H/>CH_2_ stretching due to the existence of saturated aliphatic alkenes, especially, methylene group. A medium, sharp stretching vibration positioned at 1629.16 cm^−1^ confirm the presence of C=C, R_2_C=O, –C(=O)-, N=O, –C=N-, –N=N-, R–N=N–R groups in the material has proved the existence of alkenes (olefinic alkenes-alkenyl group), organic nitrates, conjugated ketone, open-chain imino/azo group, respectively. Among these, C=C groups and C=O groups indicate the stable cyclic structure of alkenes in ring resonance and conjugated ketones, which are extensively found in secondary metabolites or bio-polymers. The following weak stretch at 1420.88 cm^−1^ is due to vinyl-C-H and R–COOH/R–CO₂H that once again proved the presence of olefinic alkenes and carboxylic acid salt/carboxylate. Likewise, the inhabitants as aromatic primary/secondary/tertiary amines, phenolic groups, organic phosphates and dialkyl/aryl sulfonates were confirmed by the small, sharp, in plane stretching noted around 1317.49 cm^−1^. The aromatic nature of the material is confirmed by the weak stretching noted at 1247.72 cm^−1^, 1101.68 cm^−1^, 1101.68 cm^−1^, 898.99 cm^−1^, and 812.81cm^-^1, which represent the Aryl-*O*-CH_2_ and Aryl-*O*-CH_3_ groups. It is also confirmed that the material possesses an epoxide or oxirane group by 1247.72 cm^−1^. Concurrent reports also demonstrated that bio-plasticizers may be epoxidized esters of fatty acids, sulfonic acid esters, diphenyl phosphates, cyclohexane dicarboxylic acid esters/cyclo-hexanoates, vinyl/aryl esters, etc. The occurrence of phenol groups and aromatic compounds point towards the existence of phenolic compounds in the sample [58]. Few halogenated groups are found in the material and is denoted by the stretching of 1101.68 cm^−1^(C–F), 1028.27 cm^−1^(P–*O*–C), 1317.49 cm^−1^(P=O), and 715.45 cm^−1^(C–Cl). Interestingly, certain specific groups were noted in the material such as organic siloxane, cyclohexane ring (1028.27 cm^−1^) and sulfonates/disulfides (1317.49 cm^−1^, 1158.34 cm^−1^, 582.99 cm^−1^). Adhesives, sealants, coatings, plastics, cosmetics, and hygiene products all benefit from organic siloxanes because of their hydrophobic, adhesive characteristics. The suspected material's organic siloxane group indicates compatibility, hydrophobicity, and plasticity. Likewise, biopolymers or bio-plasticizers belongs to the family polymerics (Example: propylene/butylene glycols, ethylene-vinyl acetate copolymers, carbon monoxide terpolymers, chlorinated polyethylene, etc.) and phosphates (Example: Alkyl diaryl phosphates) possess high molecular weight, migration resistance, solvent resistance, flame retardant, and fast fusion properties. Moreover, all the noted specific groups signify that the extracted material may be a condensed phenolic derivative that possess plasticizing effect. Table 2 explains the relationships between the FT-IR absorption bands and the chemical groups found in NLP.

**Table 2:** FT-IR bands of NLP absorption corresponding to chemical bonds.

Sl. No	Absorption Potential (cm^−1^)	Functional groups	Band strength	Probable peak range (cm^−1^)	Related chemical fractions	References
1	3338.46	O–H O–H -N-H >N–H	Strong, broad	3200–3570 3200–3400 3325–3345 3310–3360	Alcohol Polymeric compound Aliphatic primary amine Aliphatic secondary amine	[59]
2	2916.32	C–H, >CH_2_	Weak, sharper	2915–2935	Saturated aliphatic alkene (Methylene)	[60]
3	2848.49	C–H, >CH_2_	Weak, sharper	2845–2865	Saturated aliphatic alkene (Methylene)	[61]
4	1629.16	C=C R_2_C=O, –C(=O)–N=O -C=N–N=N-R-N=N–R	Medium, sharp	1600–1700 1620–1680 1620–1640 1600–1650 1590–1690 1575–1630	Alkenes Olefinic alkenes (Alkenyl group) Organic nitrates Conjugated ketone (Quinone) Open-chain imino group Open-chain azo group	[62,63]
5	1420.88	v-C-H R–COOH or R–CO₂H	Weak, in plane stretch	1410–1420 1300–1420 1200–1600	Olefinic alkenes Carboxylic acid salt (Carboxylate) Carboxylic acid, Saturated aliphatic ester	[64,65]
6	1317.49	C–N C–OH/Ph-OH P=O R–SO_2_-*O*-R, S=O	Weak	1250–1360 1310–1410 1250–1350 1300–1335	Aromatic primary/secondary/tertiary amines Phenol or tertiary alcohol bend Organic phosphates Dialkyl/aryl sulfonates (esters)	[66]
7	1247.72	Aryl-*O*-CH_2_ Aryl-*O*-CH_3_	Weak	1230–1270 ∼1250	Aromatic ethers Aromatic ethers (Epoxy/oxirane ring)	[67,68]
8	1158.34	C–N C–N R–S(=O), S=O	Weak, in plane stretch	1130–1190 1150–1210 1100–1200	Aliphatic secondary amine Aliphatic tertiary amine Sulfonates	[69,70]
9	1101.68	C–H C–F	Weak	950–1225 1000–1150	Aromatic ring (aryl group) Aliphatic fluoro compound (halogenated)	[71,72]
10	1028.27	>CH_2_ P–*O*–C N–C–H Si–*O*–Si	Strong, sharp	925–1055 990–1050 1020–1090 1020–1055	Methylene, cyclohexane ring Aliphatic phosphates Primary amine Organic siloxane	[73,74]
11	898.99	vinyl-C-H, C=CH_2_ C–H	Weak, out of plane stretch	890–915 800–900 600–1000	Olefinic alkene Aromatic 1,3 para/meta distribution Alkyl halides, glycosides	[75,76]
12	812.81	C–H	Weak	800–900 800–890	Aromatic 1,3 para/meta distribution Aromatic ethers (Epoxy/oxirane ring)	[77,78]
13	715.45	C–Cl R–S–C(=O)−R	Weak, out of plane stretch	700–800 670–715	Aliphatic chloro compound (halogenated) Aryl thio-esters (C-5)	[79,80]
14	582.99	C–S/R–S–S−R′	Weak stretch	570–705	Disulfides	[81,82]

### 3.3 UV absorption spectra of NLP

**Fig. 3:**
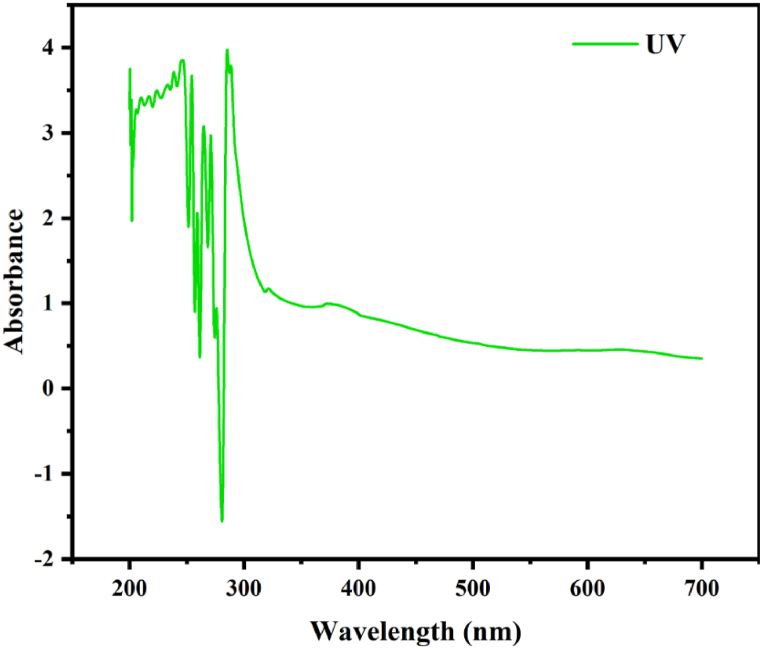
UV spectrogram of NLP.

Fig. 3 displays the spectra obtained by measuring the UV–visible absorbance of the bio-polymer sample between 200 and 700 nm. The three most prominent peaks for NLP are located at 215 nm, 275 nm, and 360 nm in wavenumber space. The absorbance range 200–350 indicates the presence of bio-active compounds like flavonoids and other phenolic compounds. Specifically, the absorbance at 215 nm could be ascribed by gallic acid or 3,4,5-trihydroxybenzoic acid and 275 nm is attributed by the bioactive compound flavan-3-ol or catechol, which belongs to the subgroup of polyphenols called flavonoids. This is also supported by another study that reported the absorbance in the range of 230–290 nm indicates that the extract of *L. sativum* contains phenolic compounds such as flavonoids and tanninswhich will act as plasticizing agents [83,84]. The absorbance at 350 nm is attributed by the flavonoid quercetin, as reported by Contini et al., in 2008 [85]. The unsaturated aliphatic/aromatic groups and heteroatoms as S, N and O of the sample were detected by the appearance of more peaks in the absorption region of 200–400 nm. The similar trend is realized through FT-IR spectra that revealed the existence of amino, sulphide and hydroxyl groups in the NLP. On the other hand, the plasticizing nature of the NLP has been confirmed by comparing the similar absorbance rate of NLP with that of other plasticizers or related compounds that have already been established. The compounds that show plasticizing property like diethyl hexyl phthalate (210 nm, 278 nm), bio-plastics from Amorphophallus paeonifolius (350 nm) and Manihot esculenta (285 nm), dibutyl phthalate (215 nm, 278 nm, 325 nm), dioctyl phthalate (212 nm, 278 nm) and bio-plastic poly lactic acid (280 nm) revealed similar absorbance trend as reported in earlier. Conversely, the bioactive chemical fractions as phenols (274 nm, 280 nm), nitromethane (275 nm), phenyl arsol (274 nm), phenylstibole (216 nm, 283 nm), 1,3 butadiene (217 nm), 1,4 dioxane (215 nm), unsaturated ketones (217 nm), amido group (214 nm), nitro/nitrate group (270 nm, 280 nm), methylamine (215 nm), carbonyl group (186–280 nm), etc. were also detected from the organic materials or plant extracts using UV spectroscopy as well. Furthermore, FT-IR and UV–visible spectra indicate that NLP is a derivative of phenolic compounds due to the presence of chemical fractions characteristic of phenolic derivatives, such as phenols, alkenes, alkyl halides, amines, aromatics, nito-compounds, and alcohols.

### 3.4 X-ray diffraction analysis

**Fig. 4:**
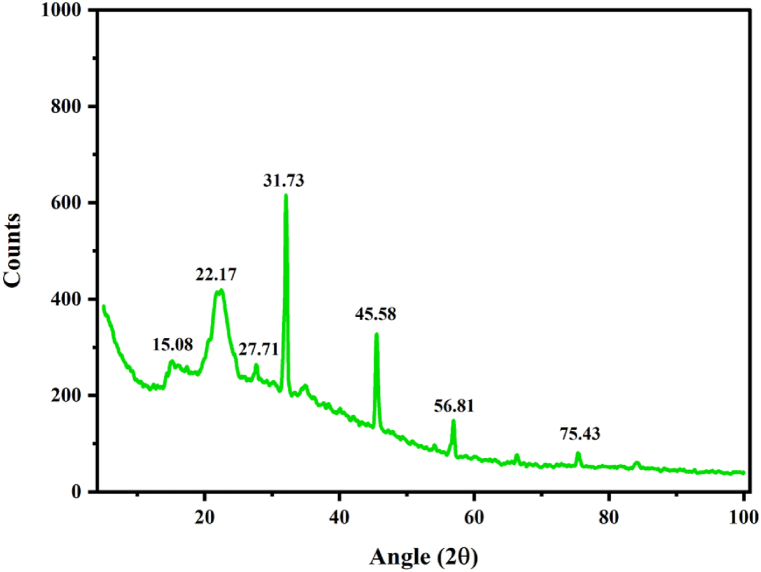
XRD spectra of NLP.

The X-ray diffraction (XRD) spectra of NLP are depicted in Fig. 4. The amorphous and crystalline patterns are seen as broad and sharp peaks, respectively. Seven prominent peaks were noted in the spectra such as 15.08°, 22.17°, 27.71°, 31.73°, 45.58°, 56.81°, and 75.43°. Where, the prominent wide peak positioned around 22.17° indicates amorphous fraction whilst the vivid peak observed at 31.73° indicates the crystalline fraction. Similar specific peaks were noted for the bio-plasticizers made of 0.45%glycerol+ 1% gum ghatti (21.93°, 33.16°, 44.73) and starch + glycerol + acetic acid + ethanol (22.27°) [86]. The remaining peaks are less defined, which revealed that there are structural transitions occurred in the material and such peaks were absent in case of most of the primary metabolites (starch, cellulose) studied. Generally, structural transitions among polymers due to molecular interactions during condensation lead to varying functional properties, which largely affect the transition of amorphous phase to crystalline. Such structural transitions might me the reason for altering the amorphous nature and improving the mechanical properties of starch or other polymers after polymerization with other chemical groups [87].

The NLP was found to have a crystallinity index of 25.1% and a crystallite size of 16.36 nm. This finding is contemporary to earlier studies that stated bio-plasticizer possess relatively low crystallinity index in the range of 19.7–37.2% [88]. In fact, the low crystallinity index of NLP is due to the absence of highly crystalline fraction. Because of its low crystalline structure, the material is more malleable and may be easily incorporated into a variety of applications.

### 3.5 Thermal analysis of NLP

#### 3.5.1 Thermal stability and activation energy analysis

**Fig. 5:**
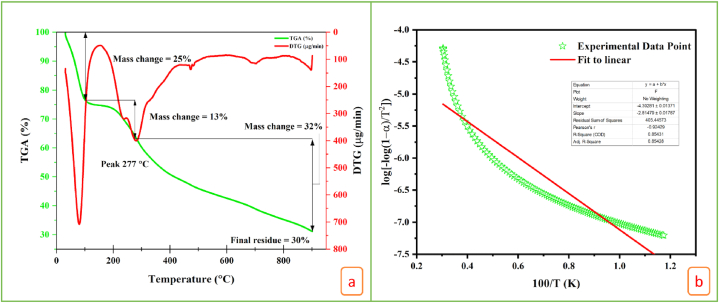
(a) TGA/DTG curves and (b) Coats-Redfern plot of NLP.

Fig. 5a, b displays the TGA/DTG profile and Coats-Redfern plot of NLP, which show the temperatures at which the material thermally degraded in a stagewise approach. Bio-plasticizer degradation was achieved in three phases via predetermined thermal analyzer treatment rates between 30 and 800 °C (Fig. 5a). TGA analysis showed signs of degradation at both the first and second stages at 96 °C, 287 °C, respectively. The TGA pattern, with first- and second-stage mass losses of 25% and 13%, is supported by the DTG curve. Bio-plasticizer moisture and volatile components were completely removed at 30 °C–96 °C. Glycols, polyphenols, and other plasticizers degraded in the second stage (230–287 °C), even though plasticizers have boiling points above 100 °C. So, the material's thermal stability is observed as 230 °C. The following heat flow degraded tannins, saponins, and other non-ferrous components in the sample (sulphur, phosphorus, etc.). The sample contained residual silica with a 30% residue [89]. This is also supported by FT-IR data that disclosed the probability of partaking siloxane in the material. Muhammed et al., in 2015 [47] reported that the plasticizing component of starch palm-based film was found to be degraded between 125 and 290 °C, whereas the degradation of starch occurred after 300 °C. Concurrent studies also disclosed related thermal stability patterns for other biopolymers such as mucilage plasticizers (176.54 °C), poly lactic acid (250 °C), starch- plasticizer films (251–313 °C), alginate- polysaccharide film (242 °C), and cellulose-glycerol blends (270–280 °C).

Coats-Redfern technique is an integral method used to calculate kinetic activation energy for thermal decomposition of the NLP, by corelating the kinetic parameters computed and protects the false-confidence in the data. It is the valid reaction model for analyzing the deviation of thermal decomposition at different heating rates against time. The best fitting line kinetic triplet from the thermo-analytical data is evaluated to imply the activation energy from decomposition rate and extent of conversion. The value obtained through this method is constant and invariant to reaction kinetics with respect to temperature alteration and degree of thermal conversion. Using the Coats-Redfern approach, in which the vast majority of the variables entered the line, we find that the kinetic activation energy of NLP is 62.20 kJ mol^−1^ (Fig. 5b). Kinetic activation energy analysis is done in this study to establish the minimum amount of energy obligatory for the thermal conversion of NLP. Table 3 compares NLP's kinetic activation energy to other bio-plasticizers. It is found that the attained kinetic activation energy is in the realistic range (44.3–93.2 kJ mol^−1^) reported for other plasticizers in earlier (Table 3).

**Table 3:** Kinetic activation energy reported for typical plasticizers along with NLP.

Bio-plasticizers	Kinetic activation energy (kJ mol^−1^)	References
Dimethyl phthalate	44.3	[48]
Propylene glycol	55.80	[90]
Diethyl phthalate	66.45	[91]
Triacetin	65.12	[90]
Glycerin	67.57	[90]
Di-*n*-butyl phthalate	78.5	[54]
Di-isobutyl phthalate	79.2	[92]
Phthalic butyl alcohol	62	[93]
Phthalic isobutyl alcohol	93.2	[94]
NLP	62.20	*This study*

#### 3.5.2 Glass transition temperature of NLP

**Fig. 6:**
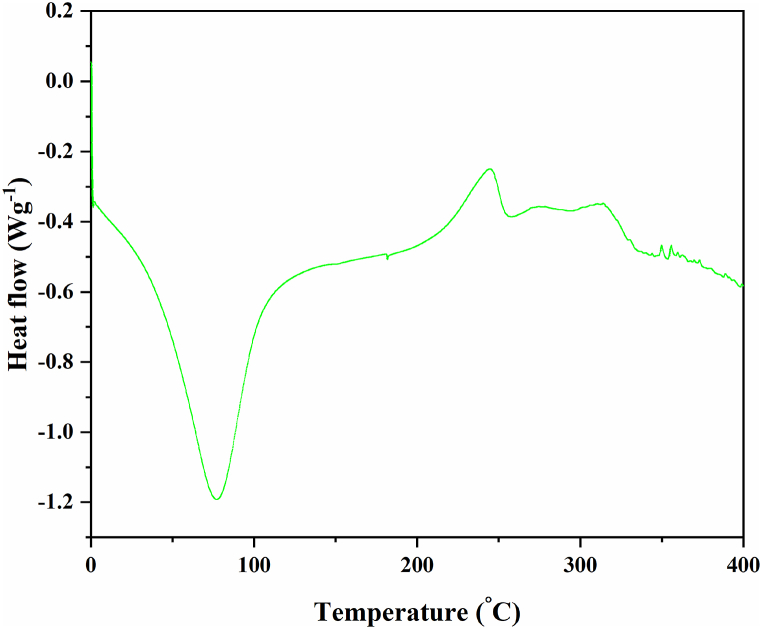
DSC analysis curve of NLP.

The DSC curve of NLP is presented as Fig. 6, of which two prominent peaks were noted in the downward and upward directions. The vivid downward peak at 77.17 °C signifies the glass transition temperature of NLP, where the primary thermal transition of the bio-polymer occurred. The following upward peak indicates the cold crystallization temperature, which is found to be 244.83 °C. The melting points of the material were assigned to be *T*m1(295.10 °C) and *T*m2 (333.42 °C). The glass transition profiles of known biopolymers with plasticizing properties, like poly lactic acid (55–72 °C), polyethylene furanoate (86 °C), poly dihydro ferulic acid (73 °C), derivatives of coumaric acid (62–95 °C), succinic acid derivative (68 °C), etc., were also reported to be comparable. According to this investigation, the extracted biopolymer possesses a glass transition temperature of 77.17 °C, which is adequate to satisfy the requirements of bio-plasticizers. Despite of high glass transition temperature of other synthetic or bio-polymers, these bio-plasticizers could be incorporated to reduce their glass transition temperature [95].

### 3.6 Morphological features of NLP

#### 3.6.1 SEM morphology and particle size

**Fig. 7:**
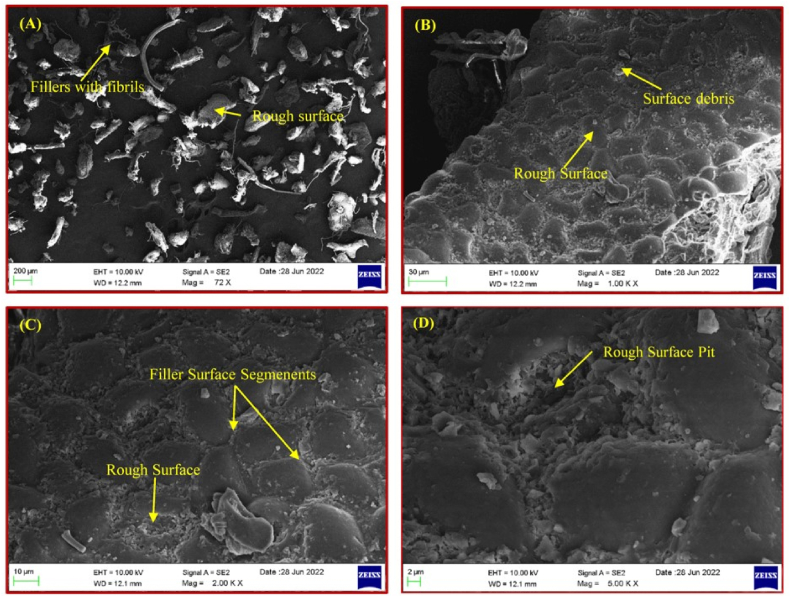
(a–d). SEM images of NLP at different magnifications.

The scanning electron microscopic images of NLP captured at different magnifications (72X, 1000X, 2000X, 5000X) are presented in Fig. 7(a–d). The figures a and b show that the individual micro-sized plasticizer particles varied in their size and shape, but fibrous in their nature. The uneven nature of the surface suggests it could offer good interfacial adhesion with other polymer matrices. Moreover, the surface of all particles is rough, since that would be a better reinforcement for biofilms and bio-composite groundwork. In order to calculate the average size of the plasticizer particles that showed varies size, ImageJ software was employed.

**Fig. 8:**
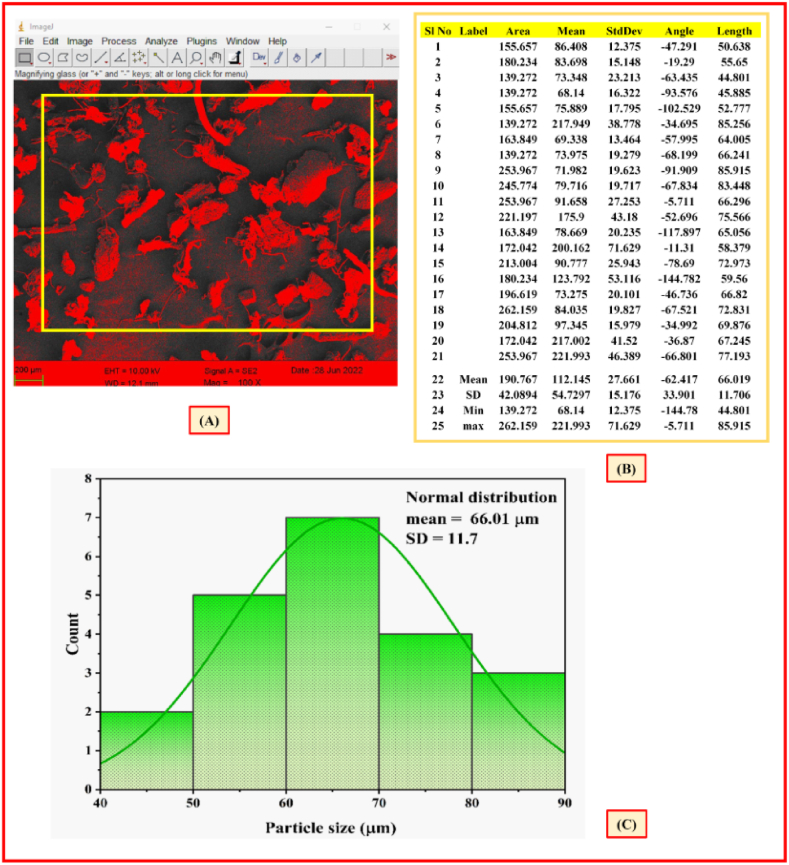
Microscopic image of particles measured (a), particle size variation of NLP (b), and size distribution histogram plot (c).

Fig. 8a-c, which shows the minimum, maximum, average, and standard deviation of the NLP particle areas. A total of 21 particles were measured in micrometres that was further used to obtain average particle size. Subsequently, the average NLP particle size was measured as 66.01 ± 11.7 μm.

#### 3.6.2 Roughness parameters of NLP

**Fig. 9:**
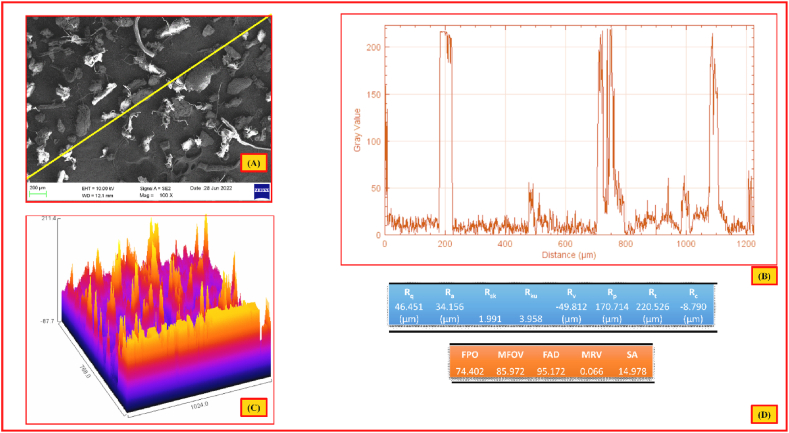
2D/3D surface view (a & b), line profile (c) and roughness parameters (d) of NLP.

Fig. 9 (a, b, c, d) shows the 2D/3D surface view (a, b), line profile (c) and roughness parameters of NLP obtained through atomic force microscopy (AFM). Like SEM images, AFM pictures (Fig. 9- a, b, c) also shows the coarseness of surface, in terms of red to orange colour patterns. The average surface roughness was calculated as 34.154 μm (R_a_) by subtracting the varied profile heights. The other roughness parameters as root mean square deviation (R_q_), surface skewness (R_sk_) and surface kurtosis (R_ku_) of NLP are found to be 46.45 μm, 1.991, 3.958, respectively. The kurtosis value of more than 3 shows that the surface is spikier, moreover, this investigation has quantitatively validated the SEM observations. Hence the extracted bio-plasticizer material was found to be suitable for fabrication of biofilm and bio-composites [96].

#### 3.6.3 Elemental dispersal of NLP

**Fig. 10:**
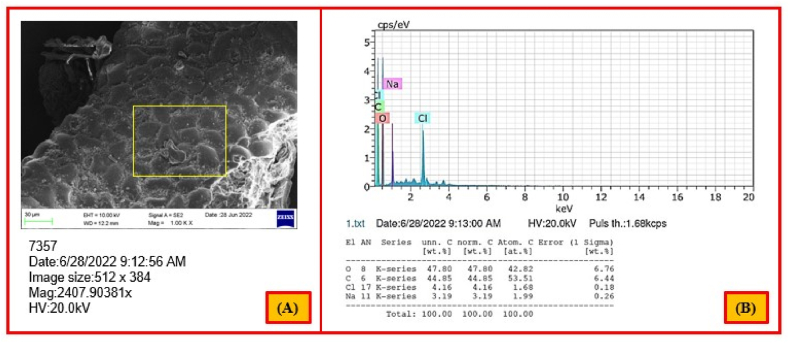
Energy-dispersive X-ray spectrogram (a) and major elements (b) of NLP.

Fig. 10 (a, b) displays the array of prominent elements present on the surface of the extracted NLP. The prime elements present on the surface were carbon (C) and oxygen (O) and the weight percentage of these elements were 47.80 wt%, 44.85 wt%, respectively. Generally, carbon and oxygen are the backbones of organic compounds, which evidenced that the material possess organic nature. Moreover, a report by Saabome in 2020 [97] ethyl acetate, a bio-plasticizer, has been shown to have a chemical structure with carbon and oxygen as its main backbones. At the same time, chlorine (Cl- 4.16 wt%) and sodium (Na- 3.19 wt%) contributed a small share in NLP. Such elements might be allied with the amine or imido or halogenated groups that associated with major pillars. The existence of these elements is also established through FT-IR analysis in this study. Although, these non-ferrous elements provide exceptional mechanical properties and good durability to the extracted bio-plasticizers from this biomass [6].

### 3.7 NLP/PLA bio-film tensile properties

**Fig. 11:**
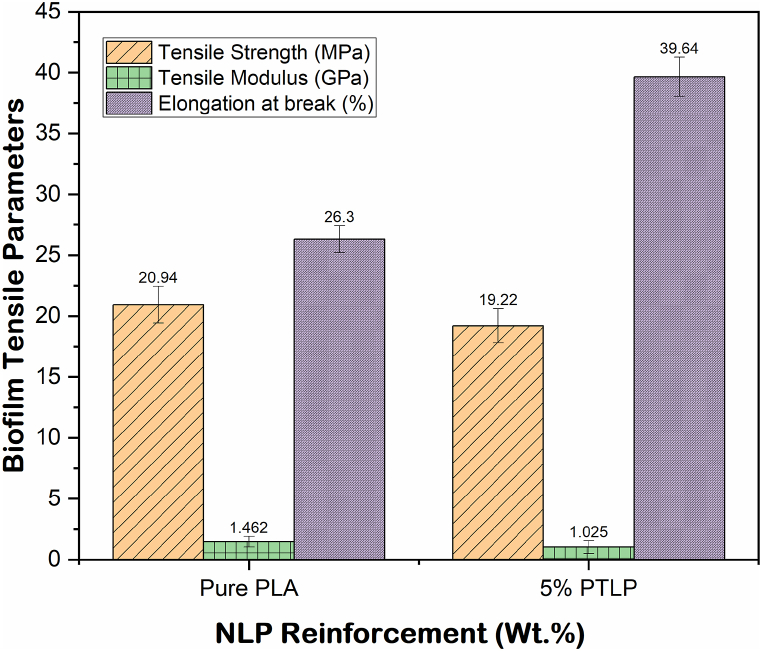
Tensile properties of pure PLA film and 5% NLP/PLA film.

Addition of plasticizer decreased tensile strength and Young's modulus and increased elongation at break (Table 4). The tensile properties of pure PLA film and NLP/PLA film are depicted in Fig. 11. The tensile strength (20.94 ± 1.5 MPa) and Young's modulus (1.462 ± 0.43 GPa) of pure PLA film was reduced to 19.22 ± 1.3 MPa and 1.025 ± 0.52 GPa after 5% NLP addition. This decreasing trend of tensile strength and Young's modulus is owed by the disruption of hydrogen bonding that causes decrease in intermolecular forces of the PLA matrix by the integration of heterocyclic groups of NLP. The tensile strength (15.24–25.5 MPa) and tensile modulus (1.17–4.6 GPa) of PLA films reported in earlier studies can correlate with that of current study [72]. In some cases, relatively higher tensile strength (35–69 MPa) was reported for pure PLA film, which is usually allied with the range of film thickness and formulation approaches [47]. Similar reducing trend in tensile strength (16.5–15.5 MPa; 35 to 22 MPa; 60.2 to 46.9 MPa) and tensile modulus (1.17–1.18 GPa; 2.35 to 1.77 GPa; 4.6 to 3.85 GPa) was observed by the addition of 1–10% plasticizers with PLA [47]. On the other hand, there is wide range of variation in tensile properties of different bio-polymers incorporated PLA films, as reported by Darie-Nita et al., in 2015 [98]. However, the percentage of elongation at break of 5% NLP/PLA film was higher (39.64 ± 1.6%) for NLP/PLA film when compared to pure PLA film (26.30 ± 1.1%). Earlier studies also stated that the addition of plasticizer to a polymer makes it more malleable and expand the utility of polymer by lifting elongation at the break. A recent study characterized corn starch (CS) plasticized fructose, glycerol, and fructose-glycerol films to understand their specific properties. Among this, glycerol (2.242–1.524 MPa), and fructose-glycerol (10.66–3.28 MPa) incorporated films showed less tensile strength as compared to NLP. The elongation at break is comparatively higher in NLP while comparing that of glycerol plasticized film (33.30–33.88%). Likewise, the elongation at break of PLA-5% N-acetyl lactate film was higher (12.8%) while comparing that of pure PLA (5.4%) film tested. The increasing trend of elongation at break of NLP is due to the plasticizing effect and lower crystallinity of the NLP incorporated. The relatively good tensile properties of NLP are owed by the good compatibility and bonding efficiency of NLP with PLA.

**Table 4:** Tensile properties of pure PLA film and 5% NLP/PLA film.

Film composites	Tensile strength (MPa)	Young’s modulus (GPa)	Elongation at break %
Pure PLA	20.94 ± 1.5	1.462 ± 0.43	26.30 ± 1.1
5% NLP/PLA	19.22 ± 1.3	1.025 ± 0.52	39.64 ± 1.6

### 3.8 NLP/PLA bio-film thermal properties

**Fig. 12:**
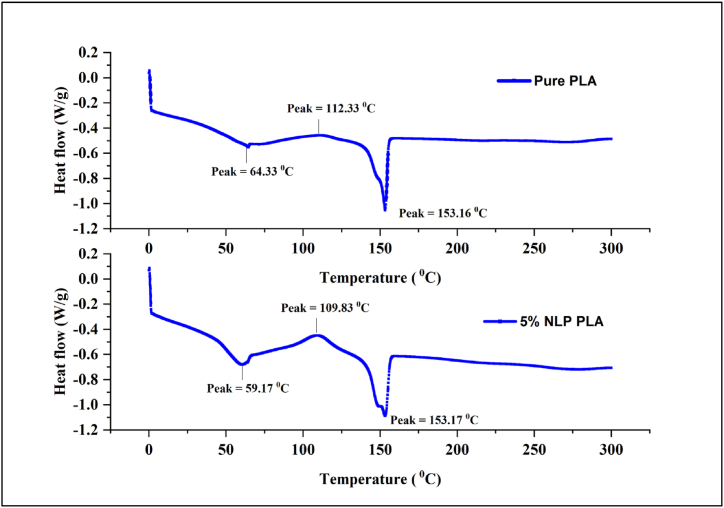
DSC spectra of pure PLA film (a) and 5% NLP/PLA film (b).

Thermal analysis of NLP/PLA film and pure PLA film through DSC analysis is necessary to conclude the variation in glass transition temperature of films and their applicability in thermal resistant, flexible packing. The resultant DSC spectra of NLP/PLA film is compared with that of PLA film in Fig. 12 (a&b). There is a decrease in glass transition temperature (Tg) from 64.33 °C (pure PLA film) to 59.17 °C (NLP/PLA film) was noted after NLP incorporation. Likewise, slight variation in cold crystallization (Tc) temperature but no change in melting temperature (Tm) was noted while comparing the spectra of both film samples. This large variation in glass transition temperature of reinforced film as compared to NLP is due to the incorporation of small NLP concentration. An earlier study revealed that a reduction of Tg from 63 °C to 41.9 °C after the addition of 10% plasticizer. Moreover, the obtained decrease in glass transition temperature is assumed by the reduced tensile strength of NLP incorporated film due to the increase in segmental mobility and decrease in intermolecular interaction of the polymer chains of PLA. Since the torque and viscosity of films decreased while adding plasticizer to the PLA. Moreover, chain deformation occurs in PLA due to frictional resistance and totally modify PLA processability. Similar variations in glass transition temperature were noted through previous studies that reported a reduction range of 60.78 °C–57.48 °C, 63.6 °C–59.9 °C, 60.42 °C–50.48 °C, 60.78 °C–52.94 °C, respectively. Likewise, plasticizers incorporated starch films showed a decrease in Tg (61.2 °C–55.8 °C) while adding 1% *Propolis* nanoparticles as plasticizers [99]. The Tg of alginate film (46 °C) was found to be decreased after incorporating it with plasticizers like lemon polysaccharide (40 °C) and fennel polysaccharide (35 °C) [100]. However, cactus mucilage-based polyols plasticized films showed low glass transition temperature in between 30 and 40 °C. However, moderate glass transition profile of 5% NLP/PLA is significant to accomplish viable temperature required processing. Such a bio-plasticizer can also be incorporated into the synthetic plastics/polymers that shows relatively higher Tg as poly ethylene (190 °C), poly vinyl chloride (350 °C), poly butadiene (210 °C), poly ethylene adipates (205 °C), poly amides (151–157 °C), poly imides (240–275 °C), etc., due to the high Tg of synthetic plastics/polymers and this could assist to improve their flexibility and application to a larger extent [101].

### 3.9 Morphology and flexibility of bio-films

**Fig. 13:**
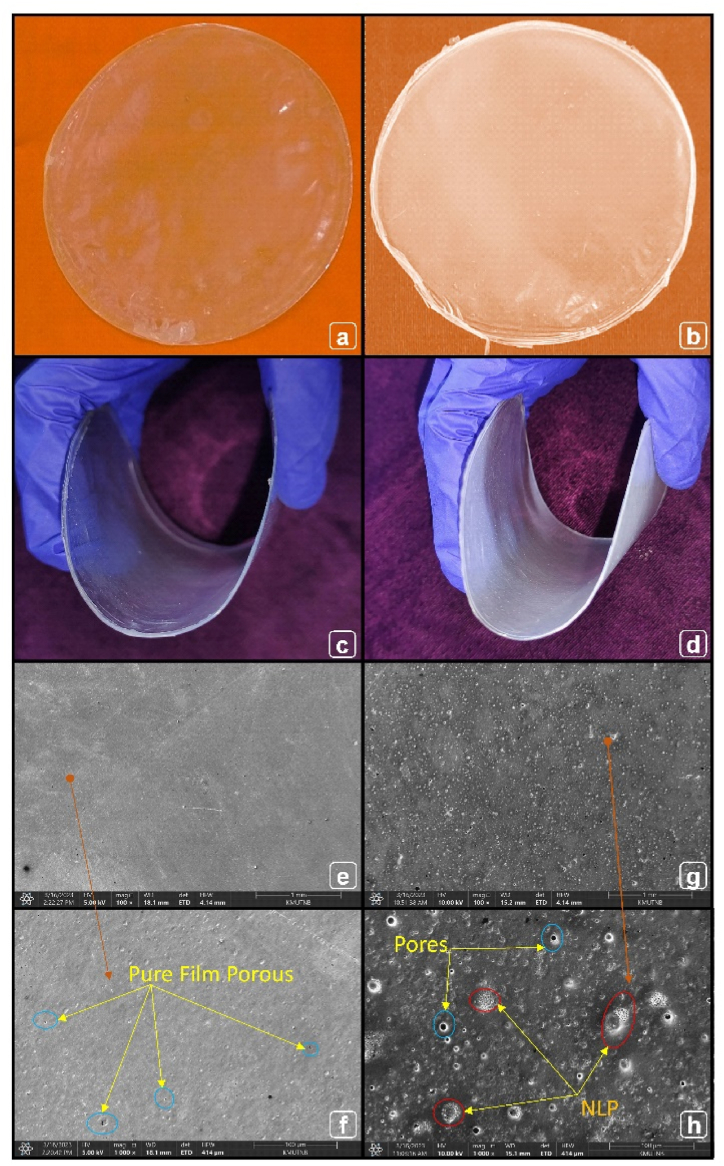
Pure PLA based bio-film (a), 5% NLP/PLA based bio-film (b), pure PLA film flexibility confirmation (c), NLP/PLA bio-film flexibility demonstrated (d), SEM images of pure PLA film (e&f) and 5% NLP/PLA based bio-film (g&h) at different magnifications.

Fig. 13 a&b displays the bio-films formulated in this study and Fig. 13 c&d illustrates the degree of flexibility of films. The visual flexibility of bio-films revealed that 5% NLP/PLA based film showed more flexibility than pure PLA film, which is due to the impact of plasticizer reinforcement. The SEM analysis of pure and plasticizer reinforced films is presented in Fig. 13 e&f, Fig. 13 g&h, respectively. The surface of pure PLA film is plane, but porous in nature with no reinforcement. The reinforcement is clearly visible in the exterior surface of the reinforced film, where less interfacial gaps are noted when compared to pure PLA film. It is convincing that the NLP fillers are sufficiently reinforced in the polymer and highly compatible with the matrix. The plasticizers are randomly oriented throughout the matrix with slight agglomeration, which is negligible. Though, optimization of reinforcement concentration is proposed for further extensive application of NLP/PLA bio-film in future.

## 4 Conclusion

Bio-plasticizers are receiving much attention today with the eventual goal of using them to develop packaging designs since that are less harmful to the environment and society. In this work, biopolymers from the *Nelumbo nucifera* leaf was extracted using chemical extraction method. The isolated biopolymers were tested for physical, chemical, thermal, and compatibility using density analysis, FT-IR, UV–Visible spectroscopy, XRD, TGA, DSC, SEM, AFM, and EDX. The occurrence of specific functional groups indicates the presence of a secondary phenolic derivative. Low crystallinity index (25.1%), low glass transition temperature (77.17 °C), good thermal stability (230 °C), low density (0.94 g/cc), and good surface roughness (R_a_-34.154 μm) suggests that the bio-plasticizer extracted is a good candidate for lightweight biofilm application. Followed by, 5% NLP reinforced bio-film and pure PLA film was formulated and characterized to establish the plasticizing and film-forming ability of NLP. NLP-incorporated films showed good surface compatibility and more flexibility than pure PLA film. The decreased tensile strength (19.22 ± 1.3 MPa), Young's modulus (1.025 ± 0.52 GPa) and glass transition temperature (59.17 °C) while increased flexibility and elongation at break (39.64 ± 1.6%) comparing with PLA film (20.94 ± 1.5 MPa, 1.462 ± 0.43 GPa, 26.30 ± 1.1%) point towards the plasticizing effect of NLP in reinforced film. The current study proposed that the obtained bio-plasticizer is a better alternative to synthetic, harmful ones for light-weight packaging applications in future.

## Declarations

### Ethical approval

Not Applicable.

## Availability of data and materials

No data was used for the research described in the article.

## CRediT authorship contribution statement

**Divya Divakaran:** Conceptualization, Data curation, Formal analysis, Funding acquisition, Investigation, Methodology, Resources, Software, Validation, Visualization, Writing – original draft, Writing – review & editing. **Malinee Sriariyanun:** Conceptualization, Data curation, Formal analysis, Investigation, Methodology, Resources, Software, Validation, Visualization, Writing – review & editing. **Indran Suyambulingam:** Conceptualization, Data curation, Formal analysis, Investigation, Methodology, Resources, Software, Validation, Visualization, Writing – original draft, Writing – review & editing. **M.R. Sanjay:** Conceptualization, Data curation, Formal analysis, Investigation, Methodology, Resources, Software, Validation, Visualization, Writing – original draft, Writing – review & editing. **Suchart Siengchin:** Conceptualization, Funding acquisition, Methodology, Project administration, Supervision, Validation, Visualization, Writing – review & editing.

## Declaration of competing interest

The authors declare that they have no known competing financial interests or personal relationships that could have appeared to influence the work reported in this paper.
